# Supplementary material for: Childhood food insecurity and incident asthma: A population-based cohort study of children in Ontario, Canada
Source: PLoS One. 2021 Jun 9;16(6):e0252301. doi: 10.1371/journal.pone.0252301 (PMC8189521; doi:10.1371/journal.pone.0252301)
Supplement: S2 Table — (DOCX) [file pone.0252301.s002.docx]

**S2 Table. Description of Ontario Health Administrative Databases**

| Database | Description |
| --- | --- |
| Ontario Registered Persons Database (RPDB) | Contains basic demographic information (age, sex, location of residence, date of birth, and date of death for deceased individuals) for those issued an Ontario health insurance number. |
| Canadian Institute for Health Information Discharge Abstract Database (DAD) | Contains administrative, clinical (diagnoses and procedures/interventions), demographic, and administrative information captured during admissions to acute care hospitals in Ontario. |
| Immigration, Refugees and Citizenship Canada Permanent Resident Database (IRCC) | Captures immigration application records for people who initially applied to land in Ontario, dating back to 1985. Records include residents' demographic information, level of education, mother tongue, and landing date. |
| National Ambulatory Care Reporting System (NACRS) | Contains administrative, clinical (diagnoses and procedures), and demographic information for all patient visits made to hospital- and community-based ambulatory care centers including emergency departments. |
| Ontario Health Insurance Plan (OHIP) | Contains information on inpatient and outpatient services provided to Ontario residents who are eligible for the province’s publicly funded health insurance system. Billing codes on the claims, identify the care provider, area of specialization, type and location of service. |
| Ontario Marginalization Index (ON-MARG) | Geographically based index that quantifies degrees of marginalization for included patients (residential instability, material deprivation, dependency, and ethnic concentration). |
| ICES Mother-Baby Linked Database (MOMBABY) | Links the DAD inpatient admission records of delivering mothers with their newborns. |
