# Supplementary material for: Childhood food insecurity and incident asthma: A population-based cohort study of children in Ontario, Canada
Source: PLoS One. 2021 Jun 9;16(6):e0252301. doi: 10.1371/journal.pone.0252301 (PMC8189521; doi:10.1371/journal.pone.0252301)
Supplement: S3 Table — (DOCX) [file pone.0252301.s003.docx]

**S3 Table. Description of the** **Household Food Security Survey Module (HFSSM)**

The HFSSM asks CCHS respondents about their experiences of food insecurity during the previous year.

Questions relate to: worrying about running out of food before there is money to buy more, cutting the size or skipping meals because there wasn’t enough money for food, going a whole day without eating because there wasn’t enough money for food

Questions target the experiences of *adults, children, and the household*.

In this study, we used the HFSSM *child scale.*  Respondents are asked about the following experiences over the previous 12 months.

- You or other adults in your household relied on only a few kinds of low-cost food to feed child(ren)
- You or other adults in your household couldn't feed child(ren) a balanced meal
- Child(ren) were not eating enough
- You or other adults in your household cut down on the size of any of the child(ren)'s meals
- Any of the child(ren) were ever hungry
- Any of the child(ren) ever skipped meals,
- Any of the child(ren) ever did not eat for whole day

Reference: The Household Food Security Survey Module (HFSSM) - Canada.ca [Internet]. [cited 2019 Sep 19]. Available from: https://www.canada.ca/en/health-canada/services/food-nutrition/food-nutrition-surveillance/health-nutrition-surveys/canadian-community-health-survey-cchs/household-food-insecurity-canada-overview/household-food-security-survey-module-hfssm-health-nutritio
