# Supplementary material for: Childhood food insecurity and incident asthma: A population-based cohort study of children in Ontario, Canada
Source: PLoS One. 2021 Jun 9;16(6):e0252301. doi: 10.1371/journal.pone.0252301 (PMC8189521; doi:10.1371/journal.pone.0252301)
Supplement: S4 Table — (DOCX) [file pone.0252301.s004.docx]

**S4 Table. Study covariates**

| **Variable** | **Database** | **Timeframe** |
| --- | --- | --- |
| Child |  |  |
| Age | RPDB | At survey completion date |
| Sex | RPDB | At survey completion date |
| Comorbidities | CIHI-DAD  NACRS  OHIP | Meets validated case definition of comorbidity from birth to survey completion date |
| Prematurity, intrauterine growth  restriction, C-section birth | DAD | At birth |
| Ethnic origin | CCHS | At survey completion date |
| Geographic location of residence (e.g.  rural or urban) | RPDB | At survey completion date |
| Healthcare utilization | OHIP | Year before survey completion date |
| Mother |  |  |
| Age | MOMBABY | At child’s birth |
| Comorbidities | CIHI-DAD  NACRS  OHIP | Meets validated case definition of comorbidity since inception of health administrative data (1991) |
| Immigration status | IRCC | At survey completion date |
| Household |  |  |
| Household food security status | CCHS | At survey completion date |
| Highest household education | CCHS | At survey completion date |
| Smoking in the home | CCHS | At survey completion date |
| Home ownership (e.g. rental) | CCHS | At survey completion date |
| Household income | CCHS and RPDB | At survey completion date |
| Number of children in household | CCHS | At survey completion date |
| Single parent household | CCHS | At survey completion date |
| Degree of marginalization | ON-MARG | At survey completion date |

Abbreviations: CCHS, Canadian Community Health Survey; CIC; Immigration, Refugees and Citizenship Canada (IRCC)’s Permanent Resident Database; DAD, Discharge Abstract Database; NACRS, National Ambulatory Care Reporting System Database; OHIP, Ontario Health Insurance Plan Database; RPDB, Registered Person’s Database of Ontario
