# Supplementary material for: Childhood food insecurity and incident asthma: A population-based cohort study of children in Ontario, Canada
Source: PLoS One. 2021 Jun 9;16(6):e0252301. doi: 10.1371/journal.pone.0252301 (PMC8189521; doi:10.1371/journal.pone.0252301)
Supplement: S5 Table — (DOCX) [file pone.0252301.s005.docx]

**S5 Table. Participant inclusions and exclusions**

| **Step** | **Inclusion/exclusion** | **n Included** | **n Excluded** | **Total n children remaining** |
| --- | --- | --- | --- | --- |
| 1 | CCHS respondents with a valid health card number & interview date | 150,218 | n/a | 150,218 |
| *Mothers* | | | | |
| 2 | Death before HFSSM interview date or missing postal code | 149,990 | 228 | 149,990 |
| 3 | Missing food security response | 149,422 | 568 | 149,422 |
| 4 | No children living in the home | 44,722 | 104,700 | 44,722 |
| 5 | Age < 18 | 31,178 | 13,544 | 31,178 |
| 6 | Mother does not have a female sex recorded | 18,016 | 13,162 | 18,016 |
| 7 | No delivery record in Ontario | 24,097 |  | 24,097 |
| *Children* | | | | |
| 8 | Death before HFSSM interview date | 23,985 | 112 | 23,985 |
| 9 | Non-Ontario Resident on Interview Date | 23,799 | 186 | 23,799 |
| 10 | Child's postal code not same as Mother's postal code on Interview Date | 20,472 | 3,327 | 20,472 |
| 11 | Age of child > =18 | 20,023 | 449 | 20,023 |
| 12 | No hospital birth record in Ontario | 8,820 | 4,724 | 8,820 |
| *Siblings* | | | | |
| 1 | Babies born to mothers included above | 17,221 |  | 17,145 |
| 2 | Child is the one who completed the CCHS | 8,455 | 8,766 | 8,437 |
| 3 | Sibling died before the HFSSM interview date | 8,379 | 76 | 8,361 |
| 4 | Sibling is a non-Ontario resident | 8,308 | 71 | 8,290 |
| 5 | Sibling's postal code not same as child's postal code on the HFSSM interview date | 6,513 | 1,795 | 6,501 |
| 6 | Sibling >=18 on interview date | 5,652 | 861 | 5,642 |
| **Combined cohort** | | | | |
| 1 | Combine eligible children, mothers, siblings | 34,495 |  | 34,337 |
| 2 | Child food security response not available | 34,318 | 177 | 34,160 |
| 3 | If > 1 food security response, keep first response | 34,042 | 0 | 34,042 |
| 4 | Prior evidence of asthma diagnosis | 27,746 | 6,296 | **27,746** |
