# Supplementary material for: Childhood food insecurity and incident asthma: A population-based cohort study of children in Ontario, Canada
Source: PLoS One. 2021 Jun 9;16(6):e0252301. doi: 10.1371/journal.pone.0252301 (PMC8189521; doi:10.1371/journal.pone.0252301)
Supplement: S6 Table — (DOCX) [file pone.0252301.s006.docx]

**S6 Table. Association between food insecurity and incident asthma** **adjusted for clinical and socioeconomic confounders**

| **Covariate** | **Adjusted Hazard Ratio (95% CI)** | | | **P value** |
| --- | --- | --- | --- | --- |
|  | **HR** | **Lower CI** | **Upper CI** |  |
| Insecure vs secure | 1.244 | 0.967 | 1.6 | 0.09 |
| Females vs males | 1.03 | 0.927 | 1.144 | 0.58 |
| Racial belonging (ref= white) ^a^ |  |  |  |  |
| Black | 1.308 | 0.932 | 1.835 | 0.12 |
| Other | 1.103 | 0.964 | 1.263 | 0.15 |
| Prematurity | 1.269 | 1.046 | 1.541 | 0.02 |
| Intrauterine growth restriction | 0.96 | 0.636 | 1.45 | 0.85 |
| GP or Pediatrician visit | 1.583 | 1.348 | 1.859 | <.0001 |
| Hospital or Emergency Department visit | 1.339 | 1.195 | 1.5 | <.0001 |
| Mother's age at child birth | 1.007 | 0.996 | 1.018 | 0.22 |
| Mother's immigration status (ref=long term resident) | 1.25 | 1.034 | 1.511 | 0.02 |
| Mother's asthma status | 1.478 | 1.279 | 1.708 | <.0001 |
| Smoking in home ^b^ | 1.056 | 0.854 | 1.306 | 0.61 |
| Deprivation quintile (ref=1) |  |  |  |  |
| Quintile 2 | 0.973 | 0.825 | 1.148 | 0.75 |
| Quintile 3 | 0.876 | 0.732 | 1.048 | 0.15 |
| Quintile 4 | 1.016 | 0.848 | 1.217 | 0.86 |
| Quintile 5 | 1.094 | 0.897 | 1.334 | 0.38 |
| Instability quintile (ref=1) |  |  |  |  |
| Quintile 2 | 0.931 | 0.791 | 1.096 | 0.39 |
| Quintile 3 | 0.949 | 0.804 | 1.12 | 0.54 |
| Quintile 4 | 0.956 | 0.804 | 1.137 | 0.61 |
| Quintile 5 | 0.909 | 0.742 | 1.114 | 0.36 |
| Rural (ref=No) | 0.883 | 0.764 | 1.02 | 0.09 |
| Home ownership (ref=Yes) ^c^ | 1.035 | 0.873 | 1.226 | 0.69 |
| Single parent household (ref=No) ^d^ | 1.09 | 0.917 | 1.296 | 0.33 |
| Household income (ref = 1st decile) |  |  |  |  |
| 2 | 1.164 | 0.895 | 1.514 | 0.26 |
| 3 | 1.231 | 0.937 | 1.619 | 0.14 |
| 4 | 1.277 | 0.972 | 1.676 | 0.08 |
| 5 | 1.441 | 1.097 | 1.893 | 0.01 |
| 6 | 1.124 | 0.846 | 1.493 | 0.42 |
| 7 | 1.165 | 0.868 | 1.562 | 0.31 |
| 8 | 1.23 | 0.912 | 1.659 | 0.17 |
| 9 | 1.119 | 0.824 | 1.518 | 0.47 |
| 10 | 1.105 | 0.782 | 1.562 | 0.57 |
| Unknown | 1.196 | 0.868 | 1.65 | 0.27 |
| Number of children in household (ref=1) |  |  |  |  |
| 2 | 0.853 | 0.751 | 0.969 | 0.01 |
| 3 | 0.704 | 0.593 | 0.835 | <.0001 |
| 4 + | 0.486 | 0.369 | 0.64 | <.0001 |
| Household education (ref=Bachelor's Degree or higher) |  |  |  |  |
| Less than secondary | 1.154 | 0.816 | 1.632 | 0.42 |
| Post-Secondary | 0.991 | 0.808 | 1.216 | 0.93 |
| Certificate | 1.144 | 0.999 | 1.309 | 0.05 |
| Unknown | 0.934 | 0.692 | 1.262 | 0.66 |

^a^ East/Southeast Asian, West Asian Arab, South Asian, and Latin American race were grouped with “Other”. ^b^ ‘Unknown’ smoking in the home was grouped with ‘no’.

Unknown’ home ownership was grouped with ‘yes’

Single-parent household was grouped with ‘no’.
