# Supplementary material for: Childhood food insecurity and incident asthma: A population-based cohort study of children in Ontario, Canada
Source: PLoS One. 2021 Jun 9;16(6):e0252301. doi: 10.1371/journal.pone.0252301 (PMC8189521; doi:10.1371/journal.pone.0252301)
Supplement: S7 Table — (DOCX) [file pone.0252301.s007.docx]

**S7 Table. Association between food insecurity and incident asthma by respondent type, adjusted for clinical confounders**

| **a. CCHS Child Self-Responders Ages 12-17 (n secure = 6257, insecure = 335)** | | | | |
| --- | --- | --- | --- | --- |
| **Covariate** | **Adjusted^a^ Hazard Ratio (95% CI)** | | | **P value** |
|  | **HR** | **Lower CL** | **Upper CL** |  |
| Insecure vs secure | 1.221 | 0.644 | 2.316 | 0.541 |
| Females vs males | 2.602 | 1.844 | 3.672 | <.0001 |
| Racial belonging (ref= white) |  |  |  |  |
| Black | 0.195 | 0.026 | 1.472 | 0.113 |
| Other | 0.704 | 0.455 | 1.090 | 0.116 |
| Prematurity | 0.615 | 0.245 | 1.545 | 0.301 |
| Intrauterine growth restriction | 1.429 | 0.518 | 3.944 | 0.491 |
| Mother's age at child’s birthday | 0.979 | 0.951 | 1.008 | 0.162 |
| Mother's immigration status (ref=long term resident) | 1.174 | 0.552 | 2.501 | 0.677 |
| Mother's asthma status | 1.221 | 0.785 | 1.899 | 0.3755 |
| Smoking in the home | 1.114 | 0.74 | 1.675 | 0.6054 |

| **b. Child not a Self-Responder Ages 0-17 (n secure = 20,066, insecure = 1079)** | | | | |
| --- | --- | --- | --- | --- |
| **Covariate** | **Adjusted^a^ Hazard Ratio (95% CI)** | | | ***P* value** |
|  | **HR** | **Lower CL** | **Upper CL** |  |
| Insecure vs secure | 1.151 | 0.892 | 1.485 | 0.280 |
| Females vs males | 0.919 | 0.822 | 1.027 | 0.135 |
| Racial belonging (ref= white) |  |  |  |  |
| Black | 1.461 | 1.033 | 2.068 | 0.032 |
| Other | 1.215 | 1.056 | 1.397 | 0.006 |
| Prematurity | 1.341 | 1.100 | 1.636 | 0.004 |
| Intrauterine growth restriction | 0.901 | 0.575 | 1.413 | 0.650 |
| Mother's age at child’s birthday | 1.005 | 0.994 | 1.017 | 0.360 |
| Mother's immigration status (ref=long term resident) | 1.237 | 1.022 | 1.496 | 0.029 |
| Mother's asthma status | 1.571 | 1.35 | 1.829 | <.0001 |
| Smoking in the home | 1.01 | 0.792 | 1.288 | 0.9356 |
