# Supplementary material for: Childhood food insecurity and incident asthma: A population-based cohort study of children in Ontario, Canada
Source: PLoS One. 2021 Jun 9;16(6):e0252301. doi: 10.1371/journal.pone.0252301 (PMC8189521; doi:10.1371/journal.pone.0252301)
Supplement: S8 Table — (DOCX) [file pone.0252301.s008.docx]

**S8 Table. Association between food insecurity and incident asthma, excluding any asthma diagnoses at ages <3 years, adjusted for clinical confounders**

| **Covariate** | **Adjusted Hazard Ratio (95% CI)** | | | ***P* value** |
| --- | --- | --- | --- | --- |
|  | **HR** | **Lower CL** | **Upper CL** |  |
| Insecure vs secure | 1.093 | 0.854 | 1.399 | 0.479 |
| Females vs males | 1.168 | 1.049 | 1.302 | 0.005 |
| Racial belonging (ref= white) |  |  |  |  |
| Black | 1.283 | 0.915 | 1.801 | 0.149 |
| Other | 1.094 | 0.954 | 1.254 | 0.198 |
| Prematurity | 1.142 | 0.925 | 1.410 | 0.215 |
| Intrauterine growth restriction | 0.886 | 0.560 | 1.400 | 0.603 |
| GP or Pediatrician visit | 1.576 | 1.350 | 1.841 | <.0001 |
| Hospital or ED visit | 1.286 | 1.146 | 1.444 | <.0001 |
| Mother's age at child birth | 1.001 | 0.990 | 1.012 | 0.842 |
| Mother's immigration status (ref=long term resident) | 1.173 | 0.969 | 1.420 | 0.101 |
| Mother's asthma status | 1.422 | 1.225 | 1.651 | <.0001 |
| Smoking in the home | 0.995 | 0.805 | 1.230 | 0.965 |

Abbreviations: ED, emergency department; GP, general practitioner
