# Supplementary material for: Childhood food insecurity and incident asthma: A population-based cohort study of children in Ontario, Canada
Source: PLoS One. 2021 Jun 9;16(6):e0252301. doi: 10.1371/journal.pone.0252301 (PMC8189521; doi:10.1371/journal.pone.0252301)
Supplement: S9 Table — (DOCX) [file pone.0252301.s009.docx]

**S9 Table. Association between childhood food insecurity and incident asthma using 3-category exposure, adjusted for clinical confounders**

| **Covariate** | **Adjusted Hazard Ratio (95% CI)** | | | **P value** |
| --- | --- | --- | --- | --- |
|  | **HR** | **Lower CI** | **Upper CI** |  |
| Moderate vs secure | 1.099 | 0.86 | 1.404 | 0.449 |
| Severe vs Secure | 2.322 | 1.095 | 4.923 | 0.028 |
| Females vs males | 1.025 | 0.923 | 1.138 | 0.644 |
| Racial belonging (ref= white) |  |  |  |  |
| Black | 1.275 | 0.909 | 1.789 | 0.159 |
| Other | 1.148 | 1.006 | 1.310 | 0.041 |
| Prematurity | 1.274 | 1.049 | 1.546 | 0.014 |
| Intrauterine growth restriction | 0.967 | 0.642 | 1.458 | 0.874 |
| Mother's age at child birth | 1.002 | 0.992 | 1.013 | 0.646 |
| Mother's immigration status (ref=long term resident) | 1.252 | 1.043 | 1.503 | 0.016 |
| Mother's asthma status | 1.518 | 1.314 | 1.754 | <.0001 |
| Smoking in the home | 1.043 | 0.847 | 1.284 | 0.6924 |

ss
