# Supplementary material for: Childhood food insecurity and incident asthma: A population-based cohort study of children in Ontario, Canada
Source: PLoS One. 2021 Jun 9;16(6):e0252301. doi: 10.1371/journal.pone.0252301 (PMC8189521; doi:10.1371/journal.pone.0252301)
Supplement: S10 Table — (DOCX) [file pone.0252301.s010.docx]

**S10 Table. Association between childhood food insecurity and incident asthma using 3-category exposure treated as continuous variable, adjusted for clinical confounders**

| **Covariate** | **Adjusted Hazard Ratio (95% CI)** | | | ***P* value** |
| --- | --- | --- | --- | --- |
|  | **HR** | **Lower CL** | **Upper CL** |  |
| Food insecurity (1: secure to 3: severely insecure) | 1.189 | 0.949 | 1.490 | 0.132 |
| Females vs males | 1.033 | 0.930 | 1.147 | 0.545 |
| Racial belonging (ref= white) |  |  |  |  |
| Black | 1.290 | 0.920 | 1.810 | 0.140 |
| Other | 1.110 | 0.972 | 1.268 | 0.123 |
| Prematurity | 1.256 | 1.034 | 1.525 | 0.022 |
| Intrauterine growth restriction | 0.968 | 0.641 | 1.461 | 0.876 |
| GP or Pediatrician visit | 1.632 | 1.391 | 1.915 | <.0001 |
| Hospital or ED visit | 1.353 | 1.209 | 1.514 | <.0001 |
| Mother's age at child birth | 1.004 | 0.993 | 1.014 | 0.491 |
| Mother's immigration status (ref=long term resident) | 1.257 | 1.047 | 1.508 | 0.014 |
| Mother's asthma status | 1.48 | 1.28 | 1.72 | <.0001 |
| Smoking in the home | 1.05 | 0.85 | 1.30 | 0.631 |

Abbreviations: ED, emergency department; GP, general practitioner
