# Supplementary material for: Childhood food insecurity and incident asthma: A population-based cohort study of children in Ontario, Canada
Source: PLoS One. 2021 Jun 9;16(6):e0252301. doi: 10.1371/journal.pone.0252301 (PMC8189521; doi:10.1371/journal.pone.0252301)
Supplement: S1 File — (DOCX) [file pone.0252301.s011.docx]

**Manuscript title: Childhood food insecurity and incident asthma: a population-based cohort study of children in Ontario, Canada**

| Study Design and Project Time Frame Definitions | | |
| --- | --- | --- |
| **Study Design** | Cohort study  Matched cohort study  Case-control study  Cross-sectional study  Other (specify): |  |
| **Project Timeline**  Look-back Window  Observation Window  (in which to look for outcomes)  **Index Event Date**  Accrual Window  Max Follow-up Date | |  |
| **Accrual Start/End Dates** | Mom Accrual: CCHS 2005, 2007-2008, 2009-2010, 2011-2012 or 2013-2014 cycles |  |
| **Max Follow-up Date** | March 31, 2018 |  |
| **When does observation window terminate?** | 1. Death 2. March 31, 2018 3. Incident asthma (ASTHMA dataset) |  |
| **Lookback Window(s)** | Look-back to 1998 |  |

| Cohort Build – CCHS Mothers & Eligible Children (Cohort 1) | | |
| --- | --- | --- |
| ***Note: Include a cohort build table in appendices.*** | | |
| **Index Event / Inclusion Criteria** | Using the CCHS 2005, 2007-2008, 2009-2010 ,2011-2012, 2013-2014 cycles, identify all individuals with   1. a valid Interview Day, Month & Year (convert to Interview Date) 2. valid IKN  - If >1 response per IKN, keep most recent one for cohort - Create variable to indicate CCHS cycle (will be necessary later).   See Appendix C for other CCHS variables to keep. | |
| **Exclusions (in order)** | *Step* | Description |
|  | 1 | Dthdate of respondent <= ***Interview Date,*** non-Ontario resident on ***Interview Date*** or Missing PSTLCODE on ***Interview Date*** |
|  | 2 | FSCE_010 or FSC_010 (CCHS variables) in (6, 7, 8, 9) |
|  | 3 | DHHEDYKD in (0, 96, 97, 98, 99) ***AND*** DHHEDOKD in (0, 96, 97, 98, 99) [Exclude no children in household] |
|  | 4 | RPDB_AGE (CCHS variable) < 18 (**NOTE:** Save these individuals in a separate dataset – see *Cohort Build – CCHS Children*) |
|  | 5 | Sex from RPDB not Female (keep only females) |
|  | 6 | No record of a delivery in MOMBABY (IKN=m_IKN) with   1. b_date <= ***Interview Date*** AND 2. B_VALIKN=”V” AND 3. WARN (from MOMBABY) = “N”   **NOTE**: From here onwards the cohort has switched to the children of the women (starting with their MOMBABY record) who responded to CCHS . Please continue to keep all the variables pulled so far, and see Appendix C for other MOMBABY variables to keep. |
|  | 7 | >1 m_key links to the same b_key (exclude these babies) |
|  | 8 | >1 record with the same B_IKN (exclude these babies)  **NOTE**: At this point, cohort should be unique on B_IKN |
|  | 9 | Dthdate of B_IKN <= ***Interview Date*** (ensure child is alive) |
|  | 10 | B_IKN non-Ontario resident on ***Interview Date*** (ensure child lives in Ontario) |
|  | 11 | PSTLCODE of B_IKN on ***Interview Date*** not same as PSTLCODE of mom’s IKN on ***Interview Date*** (ensure child lives with mom) |
|  | 12 | Age of B_IKN >= 18 on *Interview Date*  **NOTE 1**: Create a flag indicating that the CCHS response came from the mother  **NOTE 2**: Keep mother’s IKN as will be necessary for clustering analysis |

| Cohort Build – CCHS Children & Mothers (Cohort 2a) | | |
| --- | --- | --- |
| ***Note: Include a cohort build table in appendices.*** | | |
| **Index Event / Inclusion Criteria** | Individuals excluded at Step 4 from **Cohort Build – CCHS Mothers & Eligible Children** | |
| **Exclusions (in order)** | *Step* | Description |
|  | 4 | No birth record of respondent in MOMBABY (IKN=B_IKN) with   1. b_date +/- 2 months of bdate (from %getdemo) AND 2. valid m_IKN (MOMBABY variable)  - if >1 MOMBABY record, please report back.   **NOTE 1**: Please keep all the variables pulled so far, and see Appendix C for other MOMBABY variables to keep.  **NOTE 2:** Keep the mother-child pairing for the next cohort build  **NOTE 3**: Create a flag indicating that the CCHS response came from the child  **NOTE 4**: Keep mother’s IKN as will be necessary for clustering analysis |

| Cohort Build – Siblings of CCHS Children (Cohort 2b) | | |
| --- | --- | --- |
| **Index Event / Inclusion Criteria** | Starting with the mother’s (unique m_ikn) identified in Step 4 from **Cohort Build – CCHS Children & Mothers,** identify all babies born to those mothers from MOMBABY prior to the interview date.  Keep all records in MOMBABY (m_IKN) with  a) b_bdate <= Interview Date AND  b) B_VALIKN=”V” AND  c) WARN (from MOMBABY) = “N”  **NOTE**: From hereon we will be referring to the babies (B_IKN) born to these mothers. Anchor on the Children who completed the CCHS as the same siblings may be assigned to >1 response (if completed at different times by different members of the same family) | |
| **Exclusions (in order)** | *Step* | Description |
|  | 5 | >1 MOMBABY record with the same B_IKN (exclude these babies) |
|  | 6 | B_IKN = IKN of CCHS Child respondent (not a sibling) |
|  | 7 | Dthdate of B_IKN <= ***Interview Date*** (ensure child is alive) |
|  | 8 | B_IKN non-Ontario resident on ***Interview Date*** (ensure child lives in Ontario) |
|  | 9 | PSTLCODE of B_IKN on ***Interview Date*** not same as PSTLCODE of CCHS Child Respondent’s IKN on ***Interview Date*** (ensure child lives with sibling to completed CCHS) |
|  | 10 | Age of B_IKN >= 18 on *Interview Date*  **NOTE 1**: Create a flag indicating that the CCHS response came from the sibling  **NOTE 2**: Keep mother’s IKN as will be necessary for clustering analysis |

| Cohort Build – Combined | | |
| --- | --- | --- |
| ***Note: Include a cohort build table in appendices.*** | | |
| **Index Event / Inclusion Criteria** | Combine children from Cohort 1, 2a and 2b, anchoring on the interview date.  Keep the earliest interview date per child. | |
| **Exclusions (in order)** | *Step* | Description |
|  | 1 | Child food security response at *interview_date* in (6,9): Not Available / Not Specified |
|  | 2 | Prior evidence of diabetes (from ODD) |
|  | 3 | Previous solid organ transplant |
|  | 4 | If > 1 record per IKN, keep earliest record |
|  | 5 | Prior evidence of Asthma |

| Concept Definitions | | |
| --- | --- | --- |
| ***Note: Include concept definition details in appendices.*** | |  |
| **Main Exposure or Risk Factor** | Level of Childhood Food Security:   1. (0=)Food secure: No, or one, indication of difficulty with income-related food access. 2. (1=)Moderately food insecure: indication of compromise in quality and/or quantity of food consumed (2 to 4 affirmative responses) 3. (2=)Severely food insecure: indication of reduced food intake and disrupted eating patterns (>= 5 affirmative responses)   Categorize as:   1. Secure (=0) 2. Insecure (=1,2) |  |
| **Baseline Characteristics** | At Interview Date   1. Age of child (0-3, 4-5, 6-13, 14-17) 2. Sex of child 3. Income quintile 4. ONMARG – all 4 components 5. Rurality 6. Year of Response (Index Year) 7. Cultural Origin 8. Ethnic Origin   Child comorbidities (look back to birth)   1. Obesity 2. Prematurity 3. Intrauterine growth restriction / Low birthweight / Small for gestational age 4. RSV   Other Concepts   1. Mode of delivery (C section)   Health Service Utilization (look back 1 year)   1. Hospital admissions 2. ER encounters 3. GP visits 4. Pediatrician visits 5. Respirology visits   Mother’s characteristics   1. Age at child’s birth 2. Immigrant status (Refugee, Immigrant, Long-term resident)    1. NOTE: Link to IRCC; if > 1 landing date, keep the earliest one 3. Obesity 4. Charlson comorbidity score (5-year lookback) 5. Asthma   Household measures   1. Household Food Security Measure 2. Smoking in home 3. Home ownership 4. Single parent household 5. Distribution of household income – provincial level (decile) 6. Number of children in household 7. Highest level of household education   Other Concepts   - Mode of delivery (C section) |  |
| **Outcomes** | Incident Asthma (ASTHMA) |  |

| Analysis Plan and Dummy Tables **(Below is a guide – please MODIFY/EXPAND as appropriate)** |
| --- |
| Apply Asthma exclusion |
| Step 2: Obtain Baselines, Exposures, and Outcomes  1. Obtain **Asthma** in mom baseline characteristic 2. Obtain **Asthma** outcome: details provided in Appendix C, Table C4. 3. Obtain secondary **Asthma** outcome (re-coding asthma at ages <3) for sensitivity analysis (see details in Appendix Table C4) |
| Step 3: Descriptive Analyses  1. Provide number of children categorized in 3 levels of exposure (secure, moderately insecure and severely insecure) 2. Create baseline tables (Appendix B, Tab 2) reporting requested information for each baseline characteristic overall and by exposure group (two groups, secure & insecure) and p-value comparing two groups 3. Complete Other descriptors (Appendix B, Tab 2b) 4. Present outcome event rates per 1000 person years(Appendix B, Tab 3) per exposure group and unadjusted HR   **STOP HERE AND SHARE RESULTS WITH TEAM** |
| **Step 4: Main analysis – Final covariates TBD**   1. Time to event – follow children from index date to new diagnosis of asthma, censoring on death or end of follow-up to generate non-parametric Kaplan-Meier curves for new diagnosis of asthma in food secure and insecure children 2. Model 1: Generate Cox proportional hazards models to estimate HRs    1. adjust for clustering of children in same household (mother)    2. adjust for *medical* confounders: child sex, racial belonging (white, black, other – 3 categories), prematurity (binary), intrauterine growth restriction (binary), GP or Pediatrician visit (binary), hospital or ED visit (binary), mother’s age at child’s birth (continuous), mother’s immigration status (immigrant (short or longer-term) vs. long-term resident), mother’s asthma status, smoking in home    3. stratify model on birth year (see recommendation in paper below)      - 1. use age as time scale for analysis   2. generate survival curve with age as the x-axis (see paper below)      1. Model 2: Cox proportional hazards models to estimate HRs    1. adjust for clustering of children    2. adjust for medical confounders (same as Model 1)    3. adjust for additional *social* confounders: deprivation (as quintiles), instability (as quintiles), rural status, home ownership, single-parent household, household income (deciles), children in household, household education ,       - Check for collinearity, if observed (>0.5 or <-0.5), report to investigators.    4. stratify model on birth year    5. Provide correlation matrix between all covariates included in Model 2    6. use age as time scale for analysis    7. generate survival curve with age as the x-axis   **STOP: SHARE RESULTS WITH TEAM** |
| **Step 5: Sensitivity analyses** .   1. Examine for effect modification in final Model 1 (clinical model)    1. add interaction terms   Food security*sex  Food security*maternal asthma  Food security*Smoking in home  pare nested and full model using the Likelihood Ratio Test   - 1. if significant at X^2^ df=1, present keep interaction terms in final model and present HRs for these interactions (update Table 5 in manuscript)  1. Re-run Model 1 (clinical model) for secondary asthma outcome |
| 1. Complete 3 PostHoc analyses using the Medical confounders model for the Cohort study, provide Number included in complete case analyses (complete Appendix B, Tbl11). Cox PH models on the age-time scale, accounting for clustering of children within households and stratified by birth year.    1. Subgroup of CCHS child respondents only (all ages 12-17)    2. Subgroups of non CCHS respondents (all ages 0-17)    3. Use all responses, but apply a 3-level exposure (secure, moderately insecure, severely insecure) with the reference category as secure [If error due to low number of severe, make note and do not run].    4. Please conduct trend analysis and present P value for trend. To do this, re-model food security as continuous variable (1-3) and re-run adjusted models for medical confounders. P-value for food security would represent the linear trend (update Table S7 in manuscript) |

| Appendices (add appendices as needed) |
| --- |

**Appendix C: Inclusion, exclusion, baseline, and outcome definitions**

**Table C1. Inclusion/Exclusion Criteria for Cohort 1**

| **Concept** | **Data Sources** | **Code Type** |  | | **Notes**  **(including algorithm details)** |
| --- | --- | --- | --- | --- | --- |
| *Inclusion Criteria* | | | | | |
| CCHS 2005 Respondents | cchs2005_ont_link_31 | Not missing ADME_DOI, ADME_MOI & ADME_YOI |  | | **NOTE**: The 2005 CCHS has an extra ‘E’ in all variable-names, so can’t be combined with the other cycles. For cohort build, may be best rename by dropping the E, then pulling all other CCHS variables for the %codebook.  Variables to keep for cohort build:  FSCE*, RPDB_AGE, |
| CCHS 2007/2008 Respondents | cchs200708_ont_link | Not missing ADM_DOI, ADM_MOI & ADM_YOI |  | | Variables to keep for cohort build:  FSCE*, RPDB_AGE, |
| CCHS 2009/2010 Respondents | cchs200910_ont_link | Not missing ADM_DOI, ADM_MOI & ADM_YOI |  | | Variables to keep for cohort build:  FSCE*, RPDB_AGE, |
| CCHS 2011/2012 Respondents | cchs201112_ont_link | Not missing ADM_DOI, ADM_MOI & ADM_YOI |  | | Variables to keep for cohort build:  FSCE*, RPDB_AGE, |
| CCHS 2013/2014 | cchs201314_ont_link | Not missing ADM_DOI, ADM_MOI & ADM_YOI |  | | Variables to keep for cohort build:  FSCE*, RPDB_AGE, |
| Valid IKN |  | VALIKN=”V” |  | |  |
| *Exclusion Criteria* | | | | | |
| Death | RPDB |  | Ref date= Interview date |  | |
| Non-Ontario residents | RPDB |  | Ref date= Interview date |  | |
| Postal Code | RPDB |  | Ref date= Interview date |  | |
| Missing Food Security Reponses |  | FSCE_010 or FSC_010 |  | in (6, 7, 8, 9)  6 = NOT APPLICABLE 7 = DON'T KNOW 8 = REFUSAL 9 = NOT STATED | |
| Age < 18 | CCHS | RPDB_AGE |  |  | |
| Sex not Female | RPDB | Sex = “F” |  |  | |
| No delivery in MOMBABY | MOMBABY | Match on IKN=M_IKN AND b_date <= Interview Date |  | Keep following variables: B_GESTWKS_DEL, B_KEY, B_VALIKN, B_WEIGHT, B_YEAR, M_GESTWKS_DEL, M_KEY , M_IKN, M_VALIKN, WARN | |
| No Children in Household | CCHS | 2005: DHHEDYKD (No. pers. <= 15 years old in hhld), DHHEDOKD (No. pers. 16-17 years old in hhld)  Others: DHHDYKD, DHHDOKD |  | 0 = NO CHILDREN  96 = NOT APPLICABLE  97 = DON'T KNOW  98 = REFUSAL  99 = NOT STATED | |

**Table C2. Exclusion Criteria for Cohort 2a**

| **Concept** | **Data Sources** | **Code Type** | **Window** | **Notes**  **(including algorithm details)** |
| --- | --- | --- | --- | --- |
| *Exclusion Criteria* | | | | |
| No delivery in MOMBABY |  | Match on IKN=M_IKN AND b_date <= Interview Date AND B_BDATE +/- 60 days of bdate from %getdemoAND M_VALIKN=”V” |  | Keep following variables: B_GESTWKS_DEL, B_KEY, B_VALIKN, B_WEIGHT, B_YEAR, M_VALIKN, M_GESTWKS_DEL, M_KEY , M_IKN, M_VALIKN, WARN |

**Table C3. Exclusion Criteria for Combined Cohort**

| **Concept** | **Data Sources** | **Code Type** | **Window** | **Notes**  **(including algorithm details)** |
| --- | --- | --- | --- | --- |
| *Exclusion Criteria* | | | | |
| Diabetes | ODD | DIAGDATE | DIAGDATE<=Interview Date |  |
| Solid organ transplant | CORR.RECIPIENT_TREATMENT | TREATMENT_DATE | Back to Child’s BDATE (TREATMENT_DATE< Interview_Date) | If present, exclude. |
| Asthma | ASTHMA | INC_DATE | INC_DATE<Interview_date | If age at INC_DATE < 6mo, do the following:   1. If age at interview date < 1 year, recode as 0 (no asthma) 2. If age > 1 year, look for subsequent asthma diagnosis between age 1 and min (age at interview_date, 8), if found, code as 1 (positive asthma diagnosis); if not found, code as 0 (no asthma)   *OHIPDx=493 or *In DAD/SDS: ICD-9=493 or ICD-10= J45, J46 (all dx) |

**Table C3. Exposure/Baseline Characteristics**

| **Characteristic** | **Data Sources** | **Code Type** | **Window** | **Reporting Detail** | **Notes**  **(including algorithm details)** |
| --- | --- | --- | --- | --- | --- |
| Childhood Food Security | CCHS 2009-2010  2011-2012  2013-2014 | FSCDCFS2 |  | 1. Food Secure 2. Moderately food Secure 3. Severely food secure |  |
| Childhood Food Security | CCHS 2005,  2007-2008 | Derive FSCDCFS2 as per algorithm |  | 1. Food Secure 2. Moderately food Secure 3. Severely food secure |   See document with algorithm from CCHS on how to derive childhood food security levels |
| Age of child | RPDB |  | Ref date=Interview date | Age groups:  0-3, 4-5, 6-13, 14-17 |  |
| Sex of child | RPDB |  | Ref date= Interview date | N (%) each category |  |
| Income Quintile | %getdemo |  | Ref date=Interview date | 6 categories (1-5, missing) |  |
| ON-MARG | Use closest ONMARG to *interview_date* | Dependency_Q_DA  Deprivation_Q_DA  EthnicCON_Q_DA  Instability_Q_DA | Ref date=Interview date |  | Present quintiles and missing for each category |
| Rurality | %getdemo |  | Ref date=Interview date |  |  |
| Index year | CCHS |  | Ref date=Interview date | Calendar Year |  |
| Cultural/Ethnic Origin  “To which ethnic or cultural groups did ^YOUR2 ancestors belong?” | CCHS | 2005: SDCE_4[A-V]  Others: SDC_4[A-V] |  | see embedded file   | Note: ‘Yes’ where SDC_4x = 1  Individuals may fall in more than one group |
| Cultural/racial belonging  “^YOU2_C may belong to one or more racial or cultural groups on the  following list.” | CCHS | 2005: SDCE_43[A-M]  Others: SDC_43[A-M] |  | see embedded file   | Note: ‘yes’ where SDC_43x = 1  Individuals may fall in more than one group |
| Obesity | DAD |  | For both child and mother:  5 years prior to interview_date | N(%) |   See text file |
| Small for Gest  ational Age / Low Birth Weight | DAD |  | To Child’s BDATE | N(%) | See text file |
| Prematurity | DAD |  | To Child’s BDATE | N(%) | See text file |
| Hospital Admissions | DAD | AT/AP (acute hospitalizations) | 1 year prior to interview_date | Count unique episodes |  |
| ER Encounters | NACRS | Do not include scheduled | 1 year prior to interview_date | Count unique NACRS_KEY |  |
| GP visits | OHIP | SPEC code 00 | 1 year prior to interview_date | -Count only 1 claim per physician per patient per day  -Use only location=Office  -Use only FEESUFF A |  |
| Paediatric visits | OHIP | SPEC code 26 | 1 year prior to interview_date | Count only 1 claim per physician per patient per day  -Use only location=Office  -Use only FEESUFF A |  |
| Respirology visits | OHIP | SPEC code 47 | 1 year prior to interview_date | -Count only 1 claim per physician per patient per day  -Use only location=Office  -Use only FEESUFF A |  |
| Mother’s age at child’s birth | %getdemo |  | Child’s BDATE |  |  |
| Immigrant Status | IRCC (%getcic) | LANDING_DATE | LANDING_DATE < *interview_date*  If > 1 landing date per person, keep earliest one  Those with missing landing date would be categorized as long-term resident |  | Categorize as:  Recent Immigrant (<10 years)  Longer-term Immigrant (10-19 years)  Long-term resident (>=20 years or missing) |
| Charlson Comorbidity Score | DAD |  | 5 years prior to interview_date |  | Categorize as:  0  1  2+  Define those with no hospitalization as 0 |
| Household Food Insecurity | CCHS | Use FSC_* variables | Ref date=Interview date | 1. Secure 2. Marginal Food Insecurity 3. Moderate Food Insecurity 4. Severe Food Insecurity |   See document on how to code FS levels  For the purposes of the 2 by 2 table, code as 2-level variable Secure(=1) Insecure(>1)  For baseline characteristic, keep as 4-level variable |
| Someone smokes in home | CCHS | 2005: ETSE_10  Others: ETS_10 |  | Yes, No, Unknown (6-9) | 1 = YES  2 = NO  6 = NOT APPLICABLE  7 = DON'T KNOW  8 = REFUSAL  9 = NOT STATED |
| Home Ownership | CCHS | 2005: DHHE_OWN  Others: DHH_OWN |  | Yes, No, Unknown (6-9) | 1 = OWNER  2 = RENTED  6 = NOT APPLICABLE  7 = DON'T KNOW  8 = REFUSAL  9 = NOT STATED |
| Distribution of household income – provincial level | CCHS | 2005: INCEDRPR  Others: INCDRPR |  | Decile 1-10, Unknown (96-99) | 1 = DECILE 1  2 = DECILE 2  3 = DECILE 3  4 = DECILE 4  5 = DECILE 5  6 = DECILE 6  7 = DECILE 7  8 = DECILE 8  9 = DECILE 9  10 = DECILE 10  96 = NOT APPLICABLE  97 = DON'T KNOW  98 = REFUSAL  99 = NOT STATED |
| Single Parent Home | CCHS | 2005:DHHEDLVG  Others: DHHDLVG |  | Yes (5, 6, 7),  No (1, 2, 3, 4, 8, 9)  unknown (10, 96, 97, 98, 99) | 1 = UNATTACHED ALONE  2 = UNATTACHED OTHER  3 = W/SPOUSE/PARTNER  4 = PARENT SPOU/CHLD  5 = PARENT W/CHILD  6 = CHILD W/PARENT  7 = CHILD W/PAR&SIB  8 = CHILD W/2 PARENT  9 = CHLD W/2 PAR&SIB  10 = OTHER  96 = NOT APPLICABLE  97 = DON'T KNOW  98 = REFUSAL  99 = NOT STATED |
| Number of children living in household | CCHS | 2005: DHHEDYKD +, DHHEDOKD  Others: DHHDYKD + DHHDOKD |  | 1, 2, 3, 4+ | If either variable in (96, 97, 98, 99), recode to 0 (shouldn’t’ be any such cases) |
| Highest level of household education | CCHS | 2005: EDUEDH10  Others: EDUDH10 |  | see embedded file   |  |
| Mother’s Asthma Status | ASTHMA | PREV_DATE | PREV_DATE<=Interview_date |  |  |
| RSV | DAD |  | BDATE (birth date) <=DDATE<=Interview_date |  | *PLS CHECK WITH ASSIGN |
| C-section delivery | DAD, OHIP | DDATE (discharge date) for ICD-9, ICD-10  PRDATE for CCP, CCI  SERVDATE for OHIP FEECODE | ICD: -2 days to +30 days of BDATE (birth date) – would be recorded on mom’s record  CCI/CCP, OHIP: -2 days to +2 days of BDATE |  | *PLS CHECK WITH ASSIGN |

**Table C4. Outcome Definition**

| **Concept** | **Data Sources** | **Code Type** | **Window** | **Reporting Detail** | **Notes**  **(including algorithm details)** |
| --- | --- | --- | --- | --- | --- |
| Asthma | ASHTMA | DIAGDATE | INC_DATE>Interview Date | Report total number (incidence) and time to Diagnosis Date (DIAGDATE-Interview Date)/365 | If age at INC_DATE < 6mo., do the following:  -> Look for subsequent asthma diagnosis between age 1 and min (age at end of follow-up, age 8), if found, code as 1; if not found, code as 0  *OHIPDx=493 or  *In DAD/SDS: ICD-10= J45, J46 (all dx)  Incidence per 1000 person-years |
| Asthma (**for sensitivity only**) | ASHTMA | DIAGDATE | INC_DATE>Interview Date | Report total number (incidence) and time to Diagnosis Date (DIAGDATE-Interview Date)/365 | If age at INC_DATE < 3yrs., do the following:  -> Look for subsequent asthma diagnosis between age 3 and min (age at end of follow-up, age 8), if found, code as 1; if not found, code as 0  *OHIPDx=493 or  *In DAD/SDS: ICD-10= J45, J46 (all dx)  Incidence per 1000 person-years |
